# Real-world study of adebrelimab as first-line therapy for extensive-stage small cell lung cancer: a retrospective study

**DOI:** 10.3389/fimmu.2025.1678020

**Published:** 2025-10-13

**Authors:** Kaili Xu, Jian Wang, Zhenhe Weng, Junhui Wang, Jianxin Chen

**Affiliations:** ^1^ Department of Pulmonary and Critical Care Medicine, The Quzhou Affiliated Hospital of Wenzhou Medical University, Quzhou People′s Hospital, Quzhou, Zhejiang, China; ^2^ Department of Gastroenterology, Jiaxing Second Hospital, Jiaxing, Zhejiang, China; ^3^ Department of Radiation Oncology, The Quzhou Affiliated Hospital of Wenzhou Medical University, Quzhou People′s Hospital, Quzhou, Zhejiang, China; ^4^ Department of International Ward, The Quzhou Affiliated Hospital of Wenzhou Medical University, Quzhou People′s Hospital, Quzhou, Zhejiang, China

**Keywords:** adebrelimab, extensive-stage small cell lung cancer, real-world study, first-line therapy, prognostic factors

## Abstract

**Background:**

Extensive-stage small cell lung cancer (ES-SCLC) has a poor prognosis, with historical median overall survival (OS) of 8–13 months under platinum-etoposide chemotherapy. While phase III trials established adebrelimab (anti-PD-L1) plus chemotherapy as a new standard, real-world evidence remains scarce. This study evaluated real-world efficacy, safety, and prognostic factors of first-line adebrelimab-based therapy.

**Methods:**

In this retrospective study, thirty-five patients with ES-SCLC receiving adebrelimab as first-line treatment were analyzed. Endpoints included objective response rate (ORR), disease control rate (DCR), progression-free survival (PFS), OS, and adverse events (AEs). Prognostic factors were assessed via Cox regression.

**Results:**

Median age was 72 years; 88.6% were male, 85.7% had Eastern Cooperative Oncology Group Performance Status (ECOG PS) 0–1, and 51.4% had ≥2 metastatic sites. ORR was 62.8%, DCR 77.1%. Median PFS was 7.1 months (95% CI: 5.47–8.53), and median OS was 15.0 months (95% CI: 10.47–19.53). Multivariate analysis identified ECOG PS ≥2 as an independent predictor of inferior PFS (HR = 9.446, p=0.013), while ≥2 metastatic organs (HR = 3.594, p=0.046) and C-reactive Protein (CRP) ≥5 mg/L (HR = 3.337, p=0.044) predicted worse OS. Grade 3–4 AEs occurred in 74.3% of patients, primarily hematologic toxicities (neutropenia: 51.4%); two cases (5.7%) of myocarditis were observed.

**Conclusions:**

Adebrelimab suggests potentially promising efficacy in ES-SCLC, aligning with pivotal trial data despite an older cohort. ECOG PS ≥2, high metastatic burden, and elevated CRP independently predict poorer outcomes. Vigilant monitoring for hematologic toxicity and rare cardiotoxicity is warranted.

## 1 Introduction

Small cell lung cancer (SCLC) represents an aggressive neuroendocrine malignancy characterized by rapid proliferation, early metastatic dissemination, and initially high sensitivity to chemotherapy and radiotherapy, followed by almost inevitable relapse and chemoresistance (1, 2). Approximately two-thirds of patients present with extensive-stage disease (ES-SCLC) at diagnosis, historically associated with a dismal median overall survival (OS) of 8–13 months with platinum-etoposide (PE) chemotherapy alone (3, 4). Despite decades of research, therapeutic advancements for ES-SCLC remained limited until the recent integration of immune checkpoint inhibitors (ICIs) targeting the programmed death-ligand 1 (PD-L1) pathway.

The addition of PD-L1 inhibitors to first-line platinum-based chemotherapy marked a paradigm shift, demonstrating significant survival benefits in phase III trials. Atezolizumab (IMpower133 trial) and durvalumab (CASPIAN trial), both targeting PD-L1, significantly improved OS compared to chemotherapy alone, establishing the current standard of care (5, 6). More recently, adebrelimab (SHR-1316), a novel, highly selective, humanized anti-PD-L1 monoclonal antibody developed in China, demonstrated compelling efficacy in the phase III CAPSTONE-1 trial (7). When combined with carboplatin and etoposide, adebrelimab significantly prolonged both progression-free survival (PFS) and OS compared to chemotherapy alone in Chinese patients with previously untreated ES-SCLC, with a manageable safety profile (7). Based on these results, adebrelimab gained regulatory approval in China for this indication.

While randomized controlled trials (RCTs) like CAPSTONE-1 provide high-level evidence of efficacy under controlled conditions, their stringent eligibility criteria often exclude patients commonly encountered in routine clinical practice, such as older individuals, those with poorer Eastern Cooperative Oncology Group Performance Status (ECOG PS ≥2), significant comorbidities, or specific metastatic burdens (8, 9). Consequently, the real-world effectiveness and safety profile of novel therapies can differ from trial results (10). Real-world evidence (RWE) derived from observational studies is therefore crucial to complement RCT findings, offering insights into treatment patterns, outcomes, and tolerability in broader, unselected patient populations reflective of everyday oncology practice (11, 12). Such studies are particularly valuable for understanding the performance of therapies like adebrelimab in diverse clinical scenarios and identifying real-world prognostic factors (13).

Despite the established role of PD-L1 inhibitors in ES-SCLC and the approval of adebrelimab, real-world data specifically focusing on adebrelimab in the first-line setting remain scarce. To address this gap and provide valuable insights into the effectiveness, prognostic determinants, and safety of adebrelimab-based therapy outside the controlled trial environment, we conducted this retrospective, single-center, real-world study at Quzhou People’s Hospital. This study aimed to evaluate the real-world clinical outcomes, including objective response rate (ORR), disease control rate (DCR), PFS, OS, and treatment-related adverse events (AEs), in an unselected cohort of patients with ES-SCLC receiving adebrelimab as part of first-line treatment. Furthermore, we sought to identify clinical and laboratory factors associated with survival outcomes in this real-world context.

## 2 Methods

### 2.1 Study design

We retrieved data from the electronic medical record system for patients diagnosed with extensive-stage small cell lung cancer (SCLC) and treated with Adebrelimab and chemotherapy [etoposide plus cisplatin (EP), etoposide plus carboplatin (EC), or irinotecan plus cisplatin (IP)] at Quzhou People’s Hospital between September 2021 and March 2025. Patients who met the following criteria were eligible for inclusion in this retrospective real-world study: (1) a histological or cytological diagnosis of extensive-stage SCLC; (2) adequate hepatic and renal function reserve; (3) receipt of a treatment regimen that included Adebrelimab as first-line treatment; and (4) at least one measurable lesion. Additionally, the following exclusion criteria were applied: (1) a history of autoimmune disease; (2) a poor ECOG performance status score greater than 2; (3) pregnant women; and (4) multiple primary malignant neoplasms. The follow-up deadline was set for June 30, 2025. This study was approved by the Ethics Committee of Quzhou People’s Hospital, and all investigations were conducted in accordance with the Declaration of Helsinki (revised in 2013).

### 2.2 Data source and outcomes evaluations

Clinical responses were evaluated according to the Response Evaluation Criteria in Solid Tumors (RECIST) version 1.1. Radiologic assessments were performed every 2 cycles (6–8 weeks) during treatment. The objective response rate (ORR) was defined as the percentage of patients achieving a complete response (CR: complete remission of all target lesions) or a partial response (PR: at least a 30% reduction in the sum of the diameters of target lesions). Progressive disease (PD) was defined as a 20% increase in the sum of the diameters of target lesions. A disease that could not be classified as either PR or PD was evaluated as stable disease (SD). The percentage of patients with CR, PR, or SD was defined as the disease control rate (DCR). Progression-free survival (PFS) is calculated as the time from the start of adebrelimab-based treatment to the occurrence of PD or death. Overall survival (OS) is defined as the time from the start of treatment to death from any cause. Adverse events (AEs) were graded according to the National Cancer Institute Common Terminology Criteria for Adverse Events version 4.0 (NCI-CTCAE v4.0).

### 2.3 Statistical analysis

Descriptive statistics (percentages, means, and medians) were used to describe the baseline characteristics and clinical features of the extensive-stage SCLC patients. Short-term efficacy was evaluated using ORR and DCR. Survival curves were calculated using the Kaplan-Meier method and were compared via the log-rank test based on ECOG PS. K-M curves were plotted using GraphPad Prism 9.0 (GraphPad Software Inc., San Diego, CA, USA). Cut-off values for continuous variables (e.g., CRP, LDH) were determined based on established normal range. These analyses were performed using SPSS software, version 23.0 (SPSS Inc., Chicago, USA). P ≤ 0.05 was considered to indicate statistical significance.

## 3 Results

### 3.1 Patient characteristics and outcomes

A total of 35 patients were enrolled in this study. Patient characteristics are summarized in **Table 1**. The median age was 72 years. The majority of patients were male (88.6%), had a history of smoking (68.6%), and had a performance status (PS) score of 0–1 in 85.7% of cases. The distribution of metastatic organs was as follows: fewer than 2 (48.6%) and 2 or more (51.4%), with brain metastases accounting for 31.4%. No patient received prior local therapy for metastatic lesions before beginning first-line treatment. Two patients (5.7%) received thoracic radiotherapy during treatment. In this cohort, 28.6%, 51.4%, and 20% of patients received EC, EP, and IP treatments, respectively. The median number of treatment cycles for adebrelimab was 4. Additionally, the systemic inflammatory markers for patients were as follows: NLR 4.97 ± 0.64, PLR 186.08 ± 20.63, LMR 2.51 ± 0.23, PAR 5.70 ± 0.77, SII 1217.37 ± 425.90, NPR 0.04 ± 0.01, CAR 0.95 ± 0.37, CLR 34.27 ± 11.46, C-reactive Protein (CRP) 30.61 ± 10.30 mg/L, and Lactate Dehydrogenase (LDH) 329.37 ± 46.29 U/L.

**Table 1:** Baseline characteristics.

Baseline characteristics	All patients (n = 35)
Age (years), n (%)	
Median (range)	72 (52-87)
≥65	31 (88.6)
<65	4 (11.4)
Gender, n (%)	
Male	31 (88.6)
Female	4 (11.4)
Brain metastasis, n (%)	11 (31.4)
Smoking status, n (%)	
Nonsmoker	11 (31.4)
Current smoker	17 (48.6)
Former smoker	7 (20.0)
Number of metastatic organs, n (%)	
≥2	18 (51.4)
<2	17 (48.6)
ECOG PS, n (%)	
0–1	30 (85.7)
2	5 (14.3)
Adebrelimab, median (IQR)	4 (3-8)
Chemotherapy agents in the cohort, n (%)	
EP	18 (51.4)
EC	10 (28.6)
IP	7 (20.0)
Level of systemic inflammation, median (IQR)	
NLR	3.82 (2.61-6.42)
PLR	157.22 (115.72-201.59)
LMR	2.53 (1.47-3.00)
PAR	5.11 (3.50-6.30)
SII	657.80 (508.58-1158.91)
NPR	0.02 (0.19-0.03)
CAR	0.17 (0.04-0.93)
CLR	6.14 (1.13-34.41)
CPR (mg/L)	6.17 (1.55-34.83)
LDH (U/L)	244.00 (208.20-324.30)

EP, Etoposide + Cisplatin; EC, Etoposide + Carboplatin; IP, Irinotecan + Cisplatin; NLR, Neutrophil-to-Lymphocyte Ratio; PLR, Platelet-to-Lymphocyte Ratio; LMR, Lymphocyte-to-Monocyte Ratio; PAR, Platelet-to-Albumin Ratio; SII, Systemic Immune-Inflammation Index (Platelets × Neutrophils/Lymphocytes); NPR, Neutrophil-to-Platelet Ratio; CAR, C-reactive Protein-to-Albumin Ratio; CLR, C-reactive Protein-to-Lymphocyte Ratio; CRP, C-reactive Protein; LDH, Lactate Dehydrogenase.

### 3.2 Clinical outcomes

**Figure 1:**
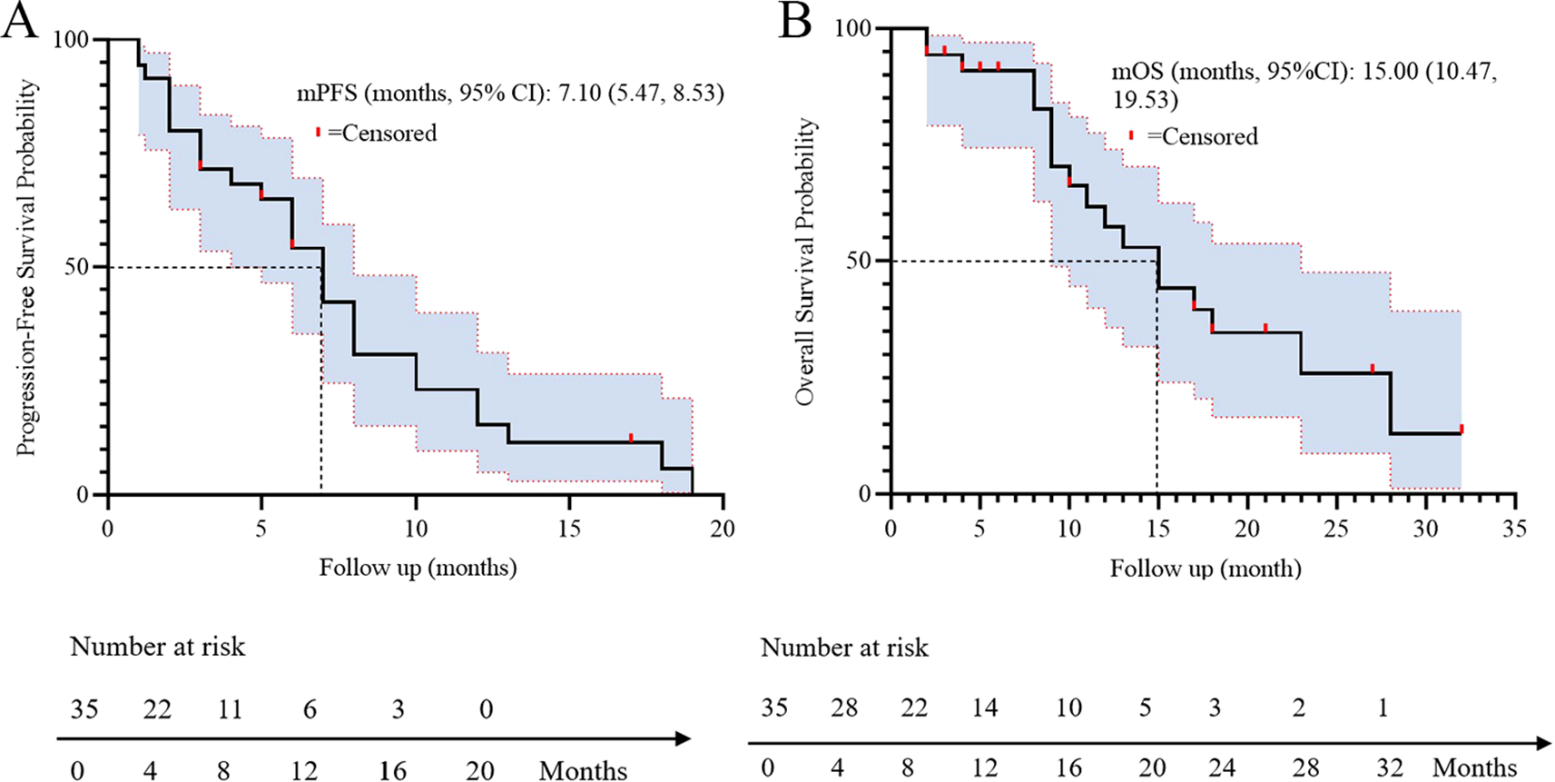
Kaplan-Meier survival curves of PFS **(A)** and OS **(B)** in 35 patients.

All patients underwent regular imaging reviews during treatment. The median follow-up time was 18.5 months (IQR: 12.3–24.1). As shown in **Table 2**, 22 of the 35 patients (62.8%) achieved a partial response (PR), 5 (14.3%) achieved stable disease (SD), and 8 (22.9%) experienced progressive disease. The disease control rate (DCR) and overall response rate (ORR) were 77.1% and 62.8%, respectively. The median progression-free survival (PFS) was 7.1 months, with a 95% confidence interval (CI) of 5.47 to 8.53 (**Figure 1A**). The median overall survival (OS) was 15.0 months, with a 95% confidence interval (CI) of 10.47 to 19.53 (**Figure 1B**).

**Table 2:** Efficacy of adebrelimab in SCLC patients (n = 35).

Efficacy	All patients (n = 35)
Complete response (%)	0
Partial response (%)	22 (62.8)
Stable disease (%)	5 (14.3)
Progressive disease (%)	8 (22.9)
Objective response rate (%, CR, PR)	22 (62.8)
Disease control rate (%, CR, PR, SD)	27 (77.1)
median progression-free survival (months, 95% CI)	7.10 (5.47, 8.53)
median Overall Survival (months, 95% CI)	15.00 (10.47, 19.53)

### 3.3 Prognostic factors for PFS and OS

**Figure 2:**
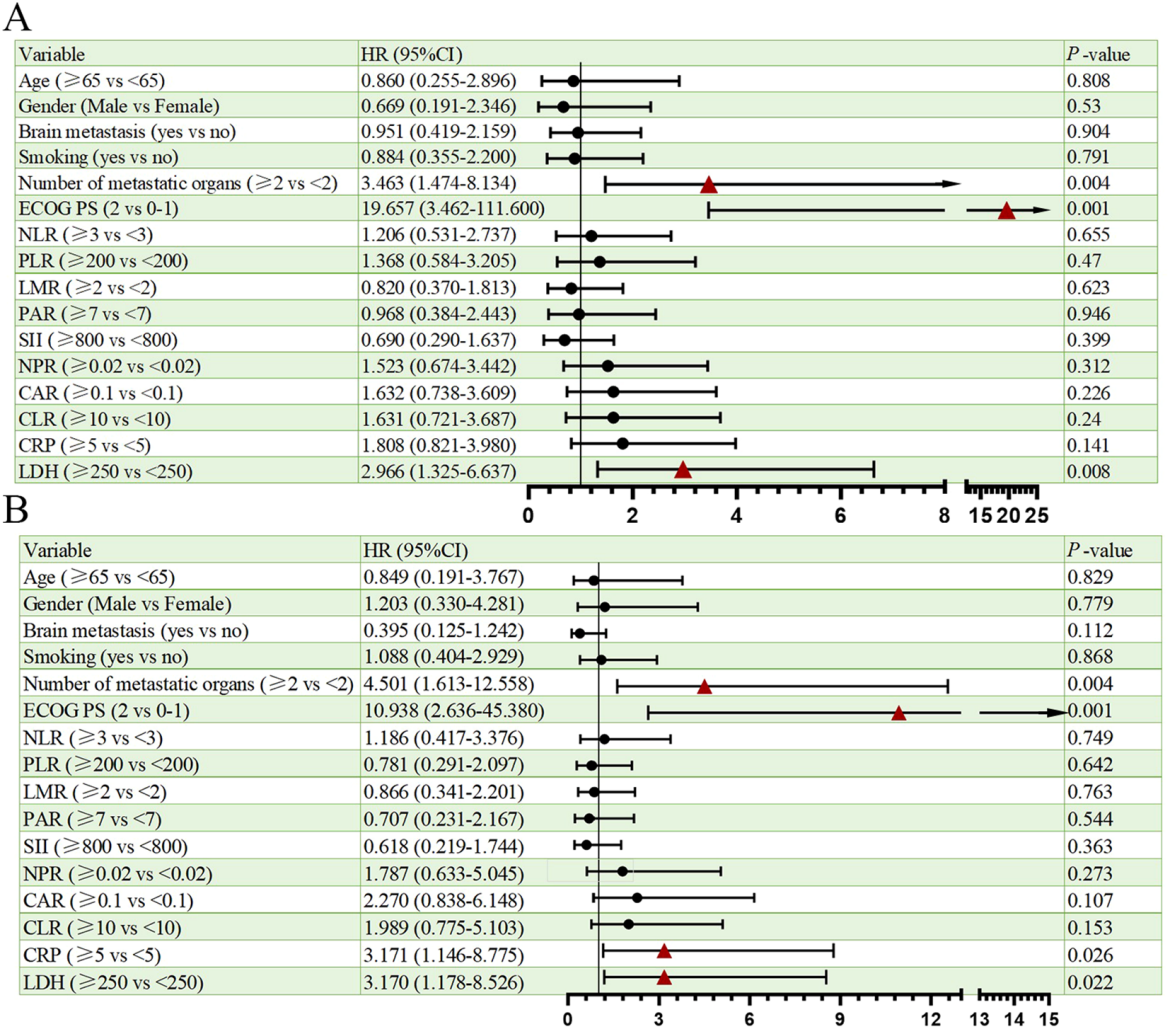
Univariate analysis of prognostic factors for PFS **(A)** and OS **(B)**.

**Figure 3:**
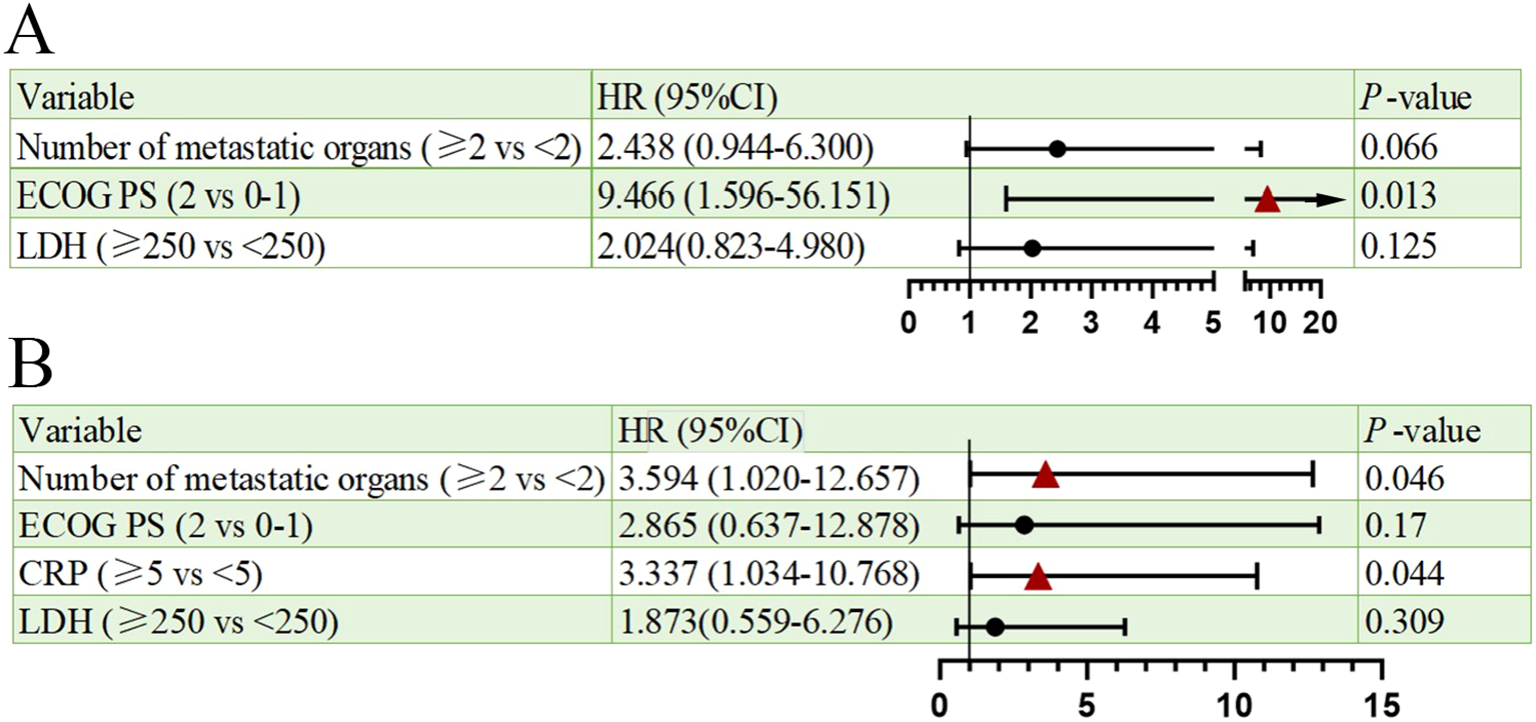
Multivariate analysis of prognostic factors for PFS **(A)** and OS **(B)**.

Univariate analysis of PFS and OS showed that the number of metastatic organs (≥2 *vs <*2; HR = 3.463, 95% CI: 1.474-8.134, p=0.004, **Figure 2A**), ECOG PS (2 *vs* 0-1; HR = 19.657, 95% CI: 3.462-111.600, p=0.001, **Figure 2A**), and LDH (≥250 *vs <*250; HR = 2.966, 95% CI: 1.325-6.637, p=0.008, **Figure 2A**) were potential risk factors for PFS. Similarly, the number of metastatic organs (≥2 *vs <*2; HR = 4.501, 95% CI: 1.613-12.558, p=0.004, **Figure 2B**), ECOG PS (2 *vs* 0-1; HR = 10.938, 95% CI: 2.636-45.380, p=0.001, **Figure 2B**), CRP (≥5 *vs <*5; HR = 3.171, 95% CI: 1.146-8.775, p=0.026, **Figure 2B**), and LDH (≥250 *vs <*250; HR = 3.170, 95% CI: 1.178-8.526, p=0.022, **Figure 2B**) were potential risk factors for OS. Furthermore, when these risk factors were incorporated into a multivariate analysis, the results demonstrated that a high ECOG PS (HR = 9.446, 95% CI: 1.596-56.151, p=0.013, **Figure 3A**) significantly influenced patients’ PFS, while CRP levels ≥ 5 (HR = 3.337, 95% CI: 1.034-10.768, p=0.044, **Figure 3B**) and the presence of ≥ 2 metastatic organs (HR = 3.594, 95% CI: 1.020-12.657, p=0.046, **Figure 3B**) were associated with a reduction in patients’ OS.

### 3.4 Subgroup analysis

**Figure 4:**
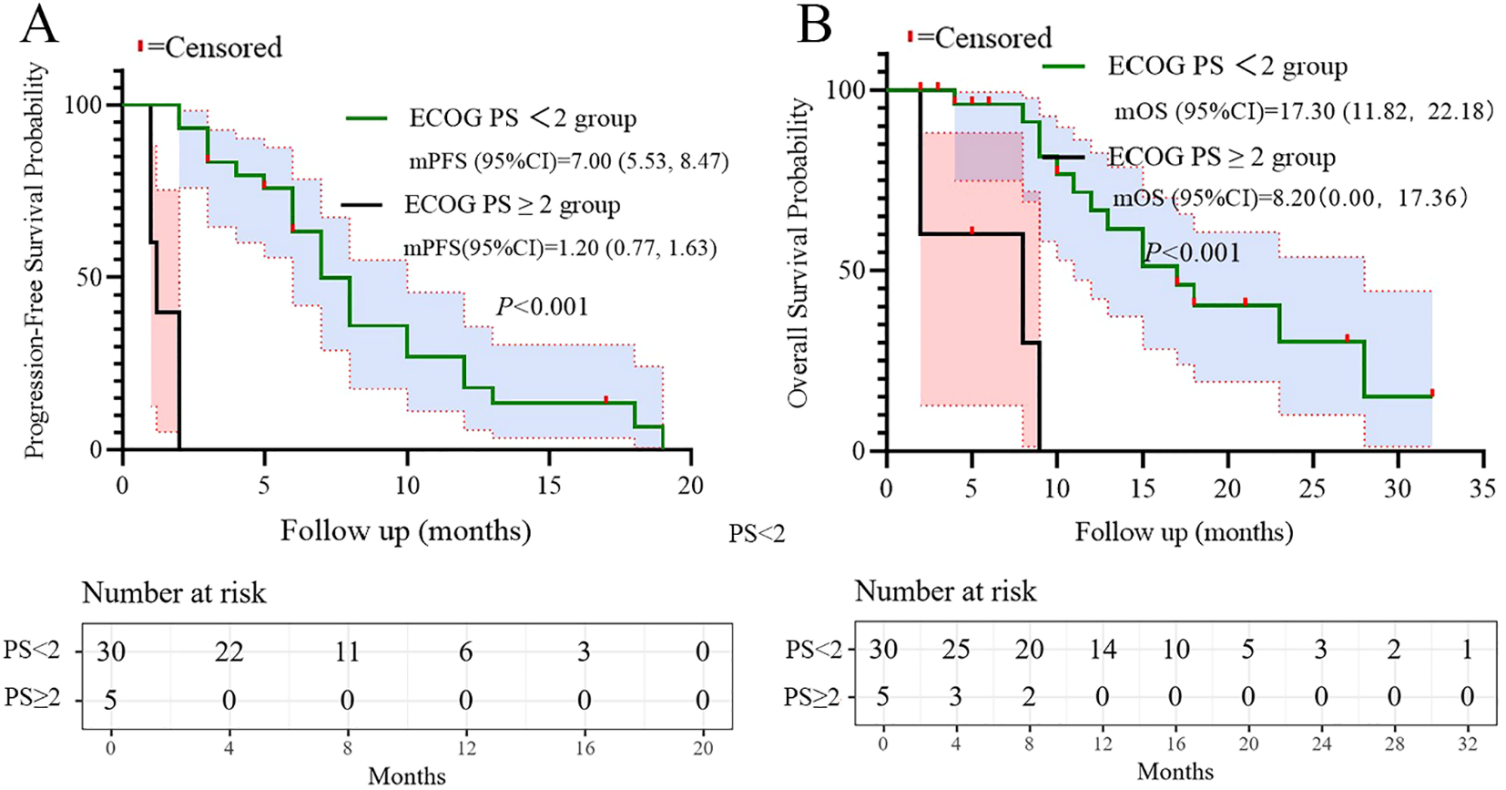
Subgroup analysis of ECOG PS as prognostic factor for PFS **(A)** and OS **(B)**.

**Figure 5:**
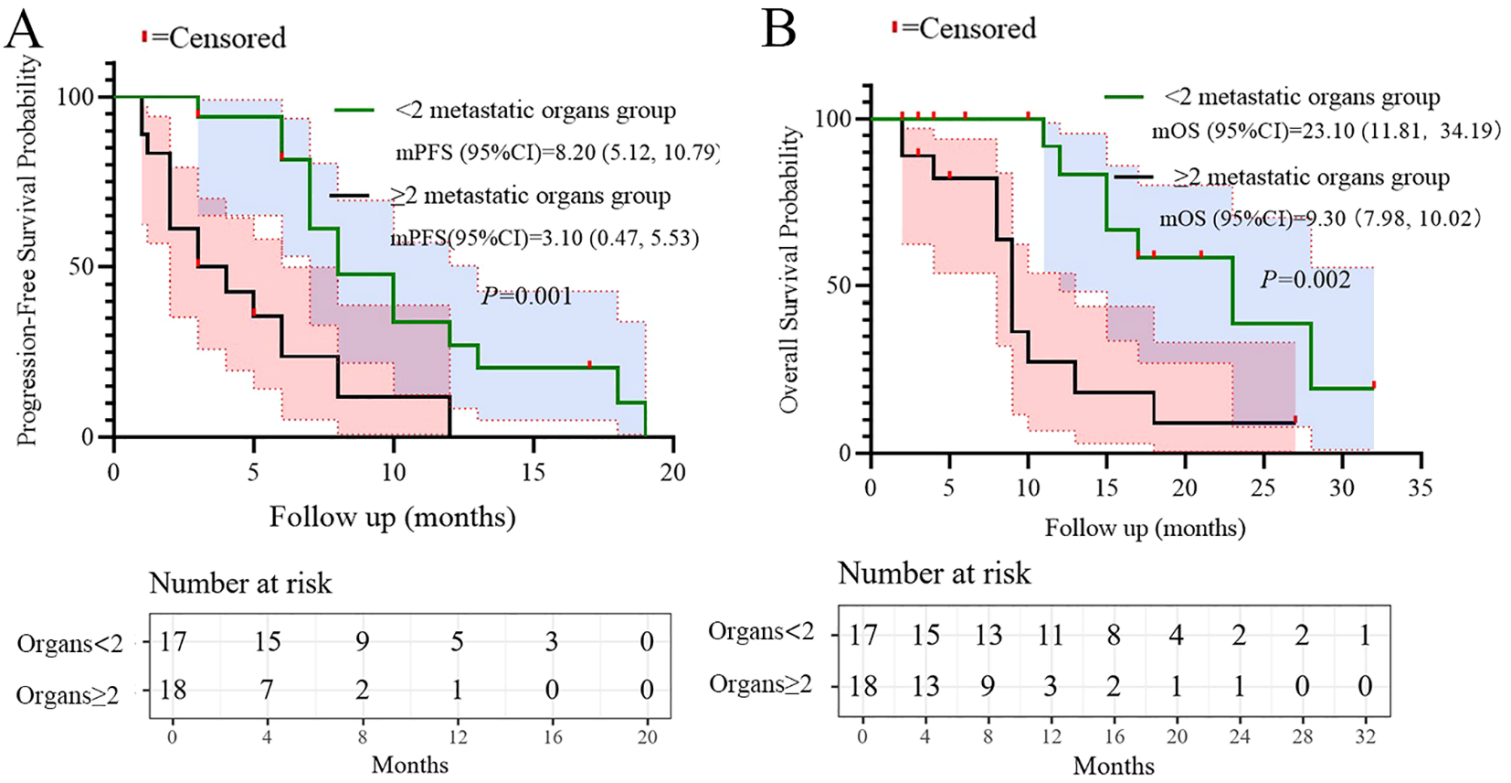
Subgroup analysis of metastatic organs as prognostic factor for PFS **(A)** and OS **(B)**.

**Figure 6:**
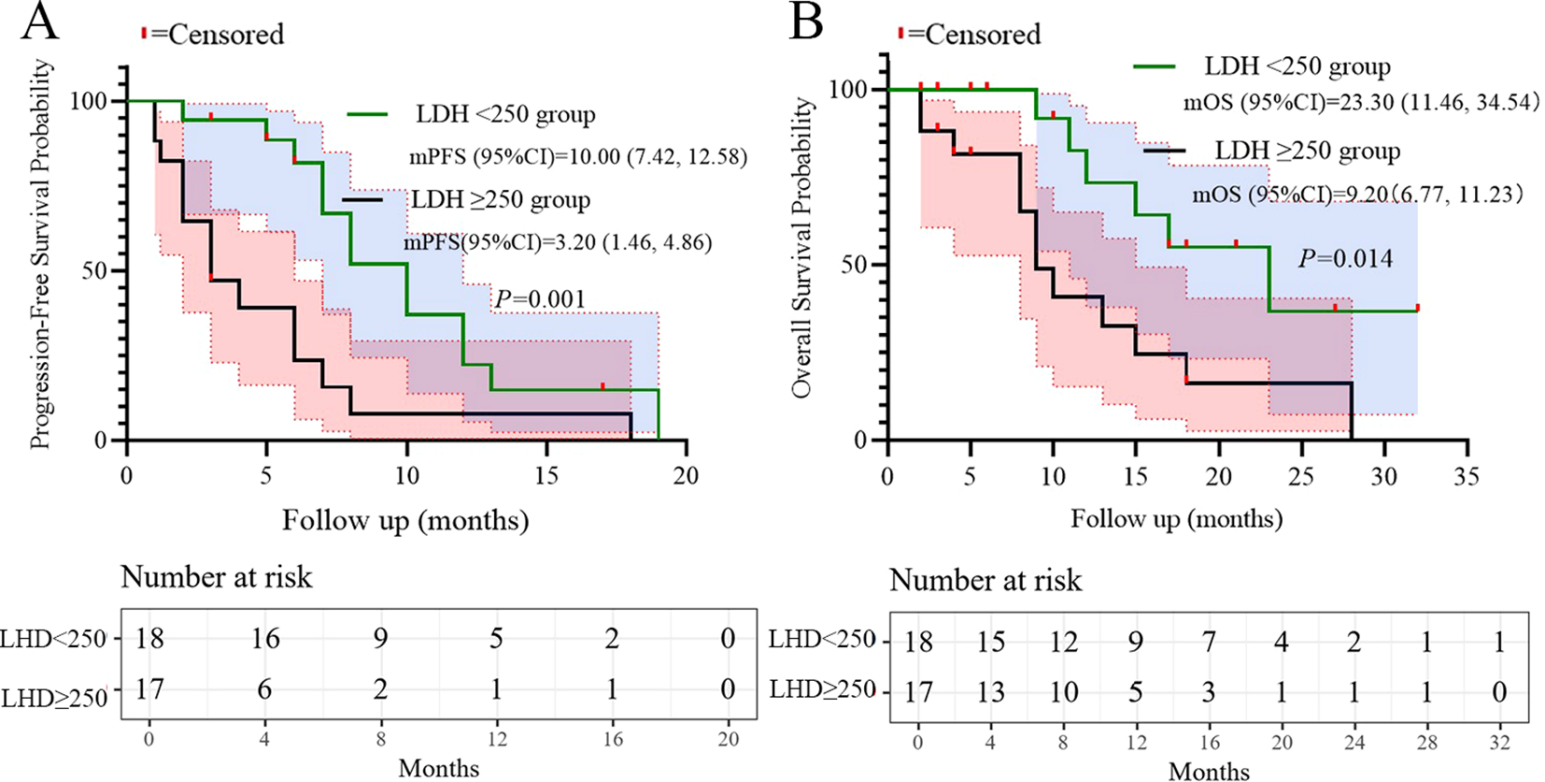
Subgroup analysis of LDH as prognostic factor for PFS **(A)** and OS **(B)**.

**Figure 7:**
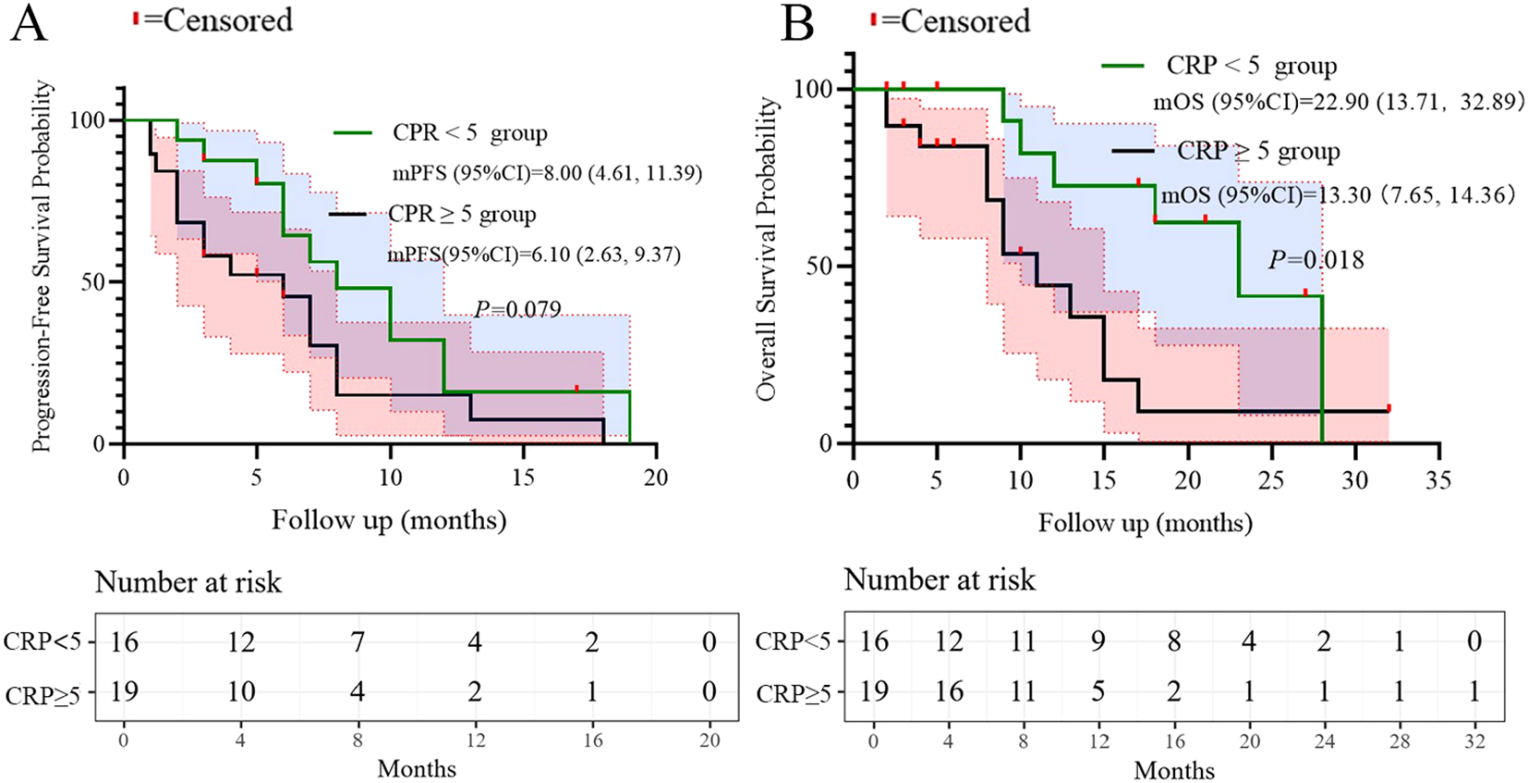
Subgroup analysis of CRP as prognostic factor for PFS **(A)** and OS **(B)**.

Based on the multivariate results, we performed subgroup analysis of PFS and OS based on CRP, LDH, PS, and the number of metastatic organs. The results showed that high PS (log-rank test p<0.001 for PFS and OS; **Figure 4**), heavier metastatic burden (log-rank test p=0.001 for PFS and p=0.002 for OS; **Figure 5**), and high LDH level (log-rank test p=0.001 for PFS and p=0.014 for OS; **Figure 6**) were independent risk factors for PFS and OS. In addition, CRP (log-rank p=0.018) was an independent risk factor for OS, but there was no statistical difference for PFS (**Figure 7**).

### 3.5 Safety

Treatment-related adverse events are summarized in **Table 3**. The most frequently reported grade 1–2 adverse events were anemia (85.7%), fatigue (74.3%), and thrombocytopenia (42.9%). Other grade 1–2 events included nausea (34.3%), neutropenia (34.3%), vomiting (28.6%), liver impairment (20.0%), elevated creatinine (11.4%), rash (8.6%), immune-related thyroiditis (8.6%), myocarditis (5.7%), and adrenal insufficiency (2.9%).

**Table 3:** Adverse events.

Adverse events	Grade 1–2, n (%)	Grade 3–4, n (%)
Anemia	30 (85.7)	5 (14.3)
Thrombocytopenia	15 (42.9)	7 (20.0)
Elevated creatinine	4 (11.4)	1 (2.9)
Fatigue	26 (74.3)	3 (8.6)
Nausea	12 (34.3)	0
Vomiting	10 (28.6)	0
Rash	3 (8.6)	0
Neutropenia	12 (34.3)	18 (51.4)
Immune-related thyroiditis	3 (8.6)	0
Immune-related adrenal insufficiency	1 (2.9)	0
Immune-related myocarditis	2(5.7)	0
Liver impairment	7 (20.0)	5 (14.3)

Grade 3–4 adverse events occurred most commonly as neutropenia (51.4%), followed by thrombocytopenia (20.0%), anemia (14.3%), liver impairment (14.3%), fatigue (8.6%), and elevated creatinine (2.9%). Notably, no grade 3–4 events were observed for nausea, vomiting, rash, or any of the immune-related adverse events. No grade 5 adverse events or treatment-related deaths were reported during the study period.

## 4 Discussion

This real-world study provides critical insights into the effectiveness, safety, and prognostic determinants of first-line adebrelimab plus chemotherapy in 35 unselected patients with ES-SCLC. Our results demonstrate a median OS of 15.0 months (95% CI: 10.47–19.53) and median PFS of 7.1 months (95% CI: 5.47–8.53), with an ORR of 62.8% and DCR of 77.1%. These outcomes align with the pivotal CAPSTONE-1 trial (OS: 15.3 months; PFS: 5.8 months) despite our cohort’s older median age (72 *vs*. 62 years) and inclusion of ECOG PS 2 patients (14.3%) (7). The slightly longer median PFS observed in our study compared to CAPSTONE-1 may be influenced by differences in assessment frequency or real-world imaging interpretation practices. The consistency underscores adebrelimab’s promising efficacy in routine practice, particularly relevant given the historically poor prognosis of ES-SCLC (median OS: 8–13 months with chemotherapy alone) (14, 15).

Multivariate analysis identified ​ECOG PS ≥2​ as an independent predictor of inferior PFS (HR = 9.446, p=0.013), while ​metastatic burden ≥2 organs​ (HR = 3.594, p=0.046) and ​CRP ≥5 mg/L​ (HR = 3.337, p=0.044) independently predicted worse OS. These findings extend prior real-world studies of PD-L1 inhibitors (16, 17). However, our novel observation that ​baseline CRP elevation​ independently correlates with survival highlights systemic inflammation’s role in SCLC progression. Elevated CRP may reflect underlying IL-6-driven inflammation, as supported by prior studies (18–20), but our study did not directly assess this mechanism. This aligns with data linking CRP to poor outcomes in ICI-treated NSCLC (21, 22). Future prospective studies incorporating serial cytokine measurements (e.g., IL-6) and tissue-based analyses are warranted to elucidate the mechanistic role of inflammatory pathways in SCLC immunotherapy resistance.

Additionally, ​LDH ≥250 U/L​ predicted poorer PFS and OS in univariate analysis (p=0.008 and p=0.022), consistent with its established role as a surrogate for high tumor metabolic activity and aggressive biology in SCLC (23). Subgroup analyses further validated that patients with high metastatic burden, ECOG PS 2, or elevated LDH had significantly shorter survival (log-rank p<0.05, **Figures 4**–**6**). These factors may aid risk stratification for personalized therapy intensification.

Grade 3–4 adverse events occurred in 74.3% of patients, predominantly hematologic toxicities: neutropenia (51.4%), thrombocytopenia (20.0%), and anemia (14.3%). This exceeds CAPSTONE-1’s grade 3–4 neutropenia rate (33.2%) (7), likely due to our cohort’s advanced age (88.6% ≥65 years) and higher comorbidity burden typical in real-world settings. The higher incidence of hematologic toxicity observed in our older real-world population may necessitate more aggressive supportive care, such as primary prophylaxis with granulocyte-colony stimulating factor (G-CSF), particularly in patients receiving concurrent chemotherapy and immunotherapy. Non-hematologic irAEs were infrequent (thyroiditis: 8.6%; myocarditis: 5.6%), mirroring CAPSTONE-1’s manageable irAE profile. However, two cases of myocarditis warrant vigilance, as cardiovascular irAEs carry high mortality in SCLC patients with smoking-related comorbidities. Proactive monitoring via troponin/ECG is recommended, especially with rising cardiotoxicity reports from PD-L1 inhibitors.

The key limitations in the present study include: ​single-center retrospective design​ with limited sample size (n=35), reducing statistical power for subgroup analyses. ​exclusion of ECOG PS >2 patients, omitting those with poorest prognosis. ​lack of PD-L1/TMB data, preventing biomarker correlation with outcomes.

The primary limitation of this study is its small sample size (n=35), which restricts the statistical power for robust multivariate modeling and increases the risk of overfitting. Therefore, the results of the multivariate analysis, including the identified prognostic factors, should be interpreted as exploratory and require validation in larger cohorts.

​In addition, heterogeneous chemotherapy backbones​ (EP/EC/IP), may introduce confounding effects, although the sample size precluded regimen-specific subgroup analysis. Future multi-center studies with larger cohorts should validate prognostic biomarkers (e.g., CRP, LDH) and explore molecular predictors (e.g., DLL3 expression, SLFN11 positivity).

## 5 Conclusions

This study suggests potential real-world effectiveness of first-line adebrelimab in ES-SCLC, achieving survival benchmarks set by RCTs despite older, comorbid patients. ECOG PS ≥2, metastatic burden ≥2 organs, and elevated CRP/LDH identify high-risk subgroups needing tailored approaches. The safety profile remains manageable, though hematologic toxicity and rare cardiotoxicity necessitate vigilant monitoring. These data support adebrelimab’s role in real-world ES-SCLC management while highlighting the critical need for biomarker-driven strategies.

## Data Availability

The original contributions presented in the study are included in the article/**Supplementary Material**. Further inquiries can be directed to the corresponding authors.
